# Dextran induces differentiation of circulating endothelial progenitor cells

**DOI:** 10.1002/phy2.261

**Published:** 2014-03-20

**Authors:** Syotaro Obi, Haruchika Masuda, Hiroshi Akimaru, Tomoko Shizuno, Kimiko Yamamoto, Joji Ando, Takayuki Asahara

**Affiliations:** ^1^ Department of Regenerative Medicine Science Tokai University School of Medicine Isehara Japan; ^2^ Vascular Regeneration Research Group Institute of Biomedical Research and Innovation Kobe Japan; ^3^ Department of Biomedical Engineering Graduate School of Medicine University of Tokyo Tokyo Japan; ^4^ Laboratory of Biomedical Engineering School of Medicine Dokkyo Medical University Tochigi Japan

**Keywords:** Culture, endothelial progenitor cell, signal transduction, transcription

## Abstract

Endothelial progenitor cells (EPCs) have been demonstrated to be effective for the treatment of cardiovascular diseases. However, the differentiation process from circulation to adhesion has not been clarified because circulating EPCs rarely attached to dishes in EPC cultures previously. Here we investigated whether immature circulating EPCs differentiate into mature adhesive EPCs in response to dextran. When floating‐circulating EPCs derived from ex vivo expanded human cord blood were cultured with 5% and 10% dextran, they attached to fibronectin‐coated dishes and grew exponentially. The bioactivities of adhesion, proliferation, migration, tube formation, and differentiated type of EPC colony formation increased in EPCs exposed to dextran. The surface protein expression rate of the endothelial markers vascular endothelial growth factor (VEGF)‐R1/2, VE‐cadherin, Tie2, ICAM1, VCAM1, and integrin αv/β3 increased in EPCs exposed to dextran. The mRNA levels of VEGF‐R1/2, VE‐cadherin, Tie2, endothelial nitric oxide synthase, MMP9, and VEGF increased in EPCs treated with dextran. Those of endothelium‐related transcription factors ID1/2, FOXM1, HEY1, SMAD1, FOSL1, NFkB1, NRF2, HIF1A, EPAS1 increased in dextran‐treated EPCs; however, those of hematopoietic‐ and antiangiogenic‐related transcription factors TAL1, RUNX1, c‐MYB, GATA1/2, ERG, FOXH1, HHEX, SMAD2/3 decreased in dextran‐exposed EPCs. Inhibitor analysis showed that PI3K/Akt, ERK1/2, JNK, and p38 signal transduction pathways are involved in the differentiation in response to dextran. In conclusion, dextran induces differentiation of circulating EPCs in terms of adhesion, migration, proliferation, and vasculogenesis. The differentiation mechanism in response to dextran is regulated by multiple signal transductions including PI3K/Akt, ERK1/2, JNK, and p38. These findings indicate that dextran is an effective treatment for EPCs in regenerative medicines.

## Introduction

Endothelial cells have ability of cell division and migration not only in embryo but also in adult life. When a part of endothelium is injured and detached, neighboring endothelial cells proliferate, migrate, and cover the exposed surface. Furthermore, endothelial cells always regenerate and new blood vessels are made in hypoxic lesions. Endothelial progenitor cells (EPCs) are also demonstrated to play an important role for the vascular regeneration (Asahara et al. 1997). EPCs are mobilized from bone marrow to peripheral blood, attach to existing endothelial cells nearby hypoxic lesions, transmigrate into tissues, proliferate, differentiate, secret angiogenic factors, and induce neovascularization (Jujo et al. 2008; Kirton and Xu 2010).

Since the discovery of EPCs, various methods to identify and isolate EPCs have been used (Fadini et al. 2008; Yoder 2009; Pearson 2010), this is because EPCs are thought to exist in the broad process of differentiation between hemangioblasts and mature endothelial cells. Recently immature EPCs are defined as circulating blood cells which form EPC colonies (Masuda et al. 2011). These colony‐forming EPCs are derived from hematopoietic stem cells (HSCs) population and express surface antigens such as CD34, CD133, vascular endothelial growth factor receptor 2 (VEGF‐R2, also called Flk1 or KDR), c‐Kit, and protein receptor tyrosine kinase, epithelial‐specific Tie2 (Asahara et al. 2011). Along with differential processes, colony‐forming EPCs lose immature markers and acquire other endothelial or monocyte markers, such as vascular endothelial cadherin (VE‐cadherin), E‐selectin, integrin αv/β3, and CD14. Then EPCs move onto a non‐colony‐forming EPC stage. These differentiating EPCs transform from circulating phenotype in suspended manner into tissue phenotype in attached manner after homing to ischemic or regenerative organs. However, the differentiation process from circulation to adhesion has not been clarified because floating‐circulating EPCs rarely attached to dishes in EPC cultures previously. The development of adhesion assay by a new technology is needed and would provide deeper insight into the molecular and physiological mechanisms at successive stages of EPC differentiation.

Dextran is a high molecular weight polymer of D‐glucose and it is produced by enzymes on the cell surface of certain lactic acid bacteria. It is used commonly to decrease vascular thrombosis. Dextran reduces the activity of factor VIII antigen (von Willebrand factor) and impairs the platelet adhesiveness (Aberg and Rausing 1978; Batlle et al. 1985; Robless et al. 2002). Dextran also increases the plasminogen activity, decreases the α2 antiplasmin activity, and inhibits the fibrin stabilization, thereby it increases the lysis of plasma clots (Carlin and Saldeen 1980; Wieslander et al. 1986a,b). Moreover, dextran is used to reduce blood viscosity and expand blood volume in bleeding. A basic research reported that dextran regulates bioactivities of endothelial cells. Dextran increased endothelial cell viability and decreased leukocyte adhesion to endothelial cells (Rouleau et al. 2010). In addition, dextran increased both protein and mRNA expression levels of intercellular adhesion molecule 1 (ICAM1) and vascular cell adhesion molecule 1 (VCAM1) and caused the nuclear translocation of nuclear factor kappa B (NFκB). These results indicate that dextran influences various molecules and cells, however, the effect of dextran on EPCs remains unknown.

In this regard, we speculate that the exposure of dextran may regulate the biology of not only mature endothelial cells but also immature EPCs derived from bone marrow microenvironments in vivo. Here, we investigated whether dextran influences biological circulating EPCs in a suspension culture. Moreover, we investigated the signal transduction pathways in response to dextran in these EPCs.

## Materials and Methods

### Materials

Dextran (MW 100,000–200,000; Sigma‐Aldrich, D4876) was weighed, dissolved in M199 medium, and filtered through a polyvinylidene difluoride membrane with a pore size of 0.45 μm (Whatman). The viscosity of dextran was measured with a rotating‐cone‐plate‐type viscometer, BIORHEOLIZER (Tokimec) at a constant temperature of 37°C. Measurements were taken over a range of shear rates to verify the Newtonian behavior of the solutions. The viscosity of 0, 5, and 10% dextran in M199 medium were 0.0108, 0.0350, and 0.0794 Poise, respectively. The osmotic pressure of dextran was measured with a cryoscopic osmometer, OSMOMAT 030 (Gonotec). The osmotic pressure of 0, 5, and 10% dextran in M199 medium were 272, 277, and 284 mOsmol/kg, respectively.

The signal transduction inhibitors used were as follows, 10 μmol/L LY294002 (Cell Signaling), 10 μmol/L PD98059 (Calbiocem, La Jolla, CA), 10 μmol/L JNK inhibitor II (Calbiocem), and 10 μmol/L SB203580 (Calbiocem).

### Isolation and preparation of human CD133‐positive cells

The protocol used in this study was approved by the Tokai University research ethics committee, Japan, and written informed consent was obtained from all participants. Human CD133‐positive cells were prepared from freshly obtained human umbilical cord blood after normal delivery as follows. Umbilical cord blood mononuclear cells were isolated by density gradient centrifugation of buffy coats using histopaque1077 (Sigma‐Aldrich, St. Louis, MO). CD133‐positive cells were purified from the mononuclear cells using anti‐CD133 monoclonal antibody‐conjugated microbeads (Miltenyi Biotec) and a magnetically activated cell sorter (auto‐MACS; Miltenyi Biotec, Bergisch Gladbach, Germany) following the manufacturer's protocol. To confirm the purity of CD133‐positive cells, the enriched CD133‐positive cells were used in cytometric analyses using a FACS Calibur flow cytometer (BD Biosciences, San Jose, CA). The isolated cells contained approximately 99% pure CD133‐positive cells. The isolated CD133‐positive cells were resuspended with freezing medium (CELLBANKER; Zenoaq) and were cryopreserved until use.

### Ex vivo suspension culture of EPCs

Freshly isolated EPCs were expanded as follows. 3×10^5^ CD133‐positive cells were cultured at 37°C under 5% CO_2_ atmosphere in Stem Span media (StemCell Technologies) with 50 ng/mL vascular endothelial growth factor (VEGF; R&D Systems, Minneapolis, MN), 20 ng/mL interleukin‐6 (R&D Systems), 100 ng/mL stem cell factor (Kirin), 20 ng/mL thrombopoietin (Wako), 100 ng/mL fms‐related tyrosine kinase 3 ligand (Wako), and 1% Penicillin/Streptomycin (Invitrogen, Carlsbad, CA) in suspended manner for 7 days. The surface protein expression rates of ex vivo expanded EPCs were analyzed by flow cytometry. The expression rates of CD133, CD34, c‐Kit, and CD31 were 40.0 ± 10.4%, 50.2 ± 12.5%, 46.9 ± 19.6%, and 88.8 ± 8.6%, respectively. To confirm the EPC activity, they were incubated with 1,1′‐dioctadecyl‐3,3,3′,3′‐tetramethylindo‐carbocyanine perchlorate‐labeled acetylated LDL (DiI‐acLDL; 1 *μ*g/mL; Biomedical Technologies, Stoughton, MA) at 37°C for 6 h. All cells showed positive staining for DiI‐acLDL. This indicates that ex vivo expanded EPCs keep approximately half of the characteristics of freshly isolated EPCs and have an endothelial phenotype. 3 × 10^4^/cm^2^ ex vivo expanded EPCs were applied on culture dishes coated with 100 μg/mL solution of human fibronectin (GIBCO, Grand island, NY), and cultured in M199 medium (GIBCO) with 5% fetal bovine serum (FBS; JRH), and EGM2 (VEGF, fibroblast growth factor‐2, epidermal growth factor, insulin‐like growth factor‐1, and ascorbic acid; Clonetics) at 37°C under 5% CO_2_ atmosphere for 7 days.

### Adhesion assay

2 × 10^4^ EPCs under a dextran‐free condition and exposed to 5% and 10% dextran for 24 h were seeded onto a human fibronectin‐coated 96‐well culture dish in M199 medium with 1% FBS. After incubation in a 5% CO_2_ incubator for 5 h, non‐adherent cells were removed by gently washing twice with phosphate buffered saline (PBS). The adhesive cells were examined under a phase contrast microscope (ECLIPSE TE300, Nikon, Yurakucho, Japan) equipped with a digital camera DSL1 (Nikon), the images (x10) were imported as JPEG files into National Institutes of Health (NIH) Image software. The number of adhesion cells was measured per each image.

### Migration assay

A modified Boyden chamber assay was performed. Using a 24 well‐transwell plate with 5 *μ*m pore size polycarbonate membranes (Corning Costar, Acton, MA), M199 medium was in the bottom chamber, and 5 × 10^4^ EPCs under a dextran‐free condition and exposed to 10% dextran for 24 h were seeded in the upper chamber coated with fibronectin. The migrated cells through the upper chamber were fixated with VECTASHIELD including 4′, 6‐diamino‐2‐phenylindole (DAPI; Vector) and were counted under a fluorescence microscope (×4) (IX70, Olympus, Shibuya, Japan).

### Proliferation assay

The cleavage amount of tetrazolium salt to formazan by cellular mitochondrial dehydrogenase was measured. 1 × 10^5^ EPCs under a dextran‐free condition and exposed to 5% and 10% dextran for 24 h were seeded to each well of fibronectin‐coated 96‐well plates and cultured in M199 medium with 5% FBS for 24 h. Thereafter, the cell proliferation assay reagent WST‐1 (Roche Applied Science, Mannheim, Germany) was added and incubated for 5 h. Absorbance at 450 nm was measured using SpectraMax 250 microplate reader (Molecular Devices, Sunnyvale, CA) and Softmax Pro (Molecular Devices).

### Tube formation

2 × 10^3^ EPCs under a dextran‐free condition and exposed to 10% dextran for 24 h and 1.5 × 10^4^ human umbilical vein endothelial cells (HUVECs) were applied in EBM‐2 medium (Lonza, Basel, Switzerland) with 2% FBS, added to an equivalent amount of Matrigel (BD Falcon) in a 96 well‐plate. After incubation at 37°C in an atmosphere of 5% CO_2_ gels were observed by using a phase contrast microscope (×4). The number of circles per tube structure was counted in each image.

### EPC colony‐forming assay

5 × 10^3^ EPCs under a dextran‐free and exposed to 10% dextran for 24 h were applied in methylcellulose‐containing M3236 medium (StemCell Technologies, Vancouver, Canada) with 20 ng/mL stem cell factor (Kirin), 50 ng/mL VEGF (R&D Systems), 20 ng/mL interleukin‐3 (Kirin), 50 ng/mL basic fibroblast growth factor (Wako), 50 ng/mL epidermal growth factor (Wako), 50 ng/mL insulin‐like growth factor‐1 (Wako), and 2 U/mL heparin (Ajinomoto) in a 3 cm‐dish. After 15 days in culture, the number of small or large type EPC colonies in a dish was counted under a phase contrast microscope.

### Real‐time PCR analysis

Total RNA samples were prepared from cells with RNeasy Mini Kit (Qiagen, Valencia, CA), and first‐strand cDNAs were generated using a PrimeScript RT reagent Kit (Takara, Ohtsu, Japan). After reverse transcription of the RNA into cDNA, real‐time polymerase chain reaction (PCR) was used to monitor gene expression with a 7500 Fast Real‐Time PCR System (Applied Biosystems, Foster City, CA) and a SDS 7900 (Applied Biosystems) according to standard procedures. PCR was performed with a TaqMan Fast Universal PCR Master Mix or SYBR Green PCR Master Mix (Applied Biosystems), and primer pairs and TaqMan probes for VEGF, purchased from Applied Biosystems, vascular endothelial growth factor receptor 1 (VEGF‐R1, also called Flt1), VEGF‐R2, VE‐cadherin, Tie2, endothelial nitric oxide synthase (eNOS), matrix metalloproteinase 9 (MMP9), inhibitor of DNA binding 1 (ID1), ID2, forkhead box m1 (FOXM1), FOXH1, hairy/enhancer of split related with yrpw motif 1 (HEY1), mothers against decapentaplegic, drosophila, homolog of, 1 (SMAD1), SMAD2, SMAD3, fos‐like antigen 1 (FOSL1), NFkB1, nuclear factor erythroid 2‐related factor 2 (NRF2), hypoxia‐inducible factor 1, alpha subunit (HIF1A), endothelial pas domain protein 1 (EPAS1), t‐cell acute lymphocytic leukemia 1 (TAL1), runt‐related transcription factor 1 (RUNX1), GATA‐binding protein 1 (GATA1), GATA2, v‐ets avian erythroblastosis virus e26 oncogene homolog (ERG), hematopoietically expressed homeobox (HHEX), and glyceraldehyde‐3‐phosphate dehydrogenase (GAPDH), respectively (Table 1). Other analyzed genes were listed as below; v‐ets avian erythroblastosis virus e26 oncogene homolog 1 (ETS1), ETS2, ets variant gene 2 (ETV2), ETV6, friend leukemia virus integration 1 (FLI1), elk3, ets‐domain protein (ELK3), e74‐like factor 1 (ELF1), ELF2, ELF3, FOXC1, FOXC2, FOXO1, FOXO3, FOXO4, FOXF1, sry‐box 7 (SOX7), SOX17, SOX18, mads box transcription enhancer factor 2, polypeptide a (MEF2A), MEF2C, GATA3, GATA4, ID3, vasculae endothelial zinc finger 1 (VEZF1), krüppel‐like c2/h2 zinc‐finger transcription factor 2 (KLF2), KLF4, KLF5, lim domain only 2 (LMO2), heart‐and neural crest derivatives‐expressed 2 (HAND2), HEY2, hairy/enhancer of split, drosophila, homolog of 1 (HES1), homeobox a9 (HOXA9), HOXB3, HOXD3, HOXD10, peroxisome proliferator‐activated receptor gamma (PPARG), aryl hydrocarbon receptor nuclear translocator (ARNT), nuclear receptor subfamily 2, group f, member 2 (NR2F2), glioma‐associated oncogene homolog (GLI1), gli‐kruppel family member 2 (GLI2), GLI3, transcription factor 4 (TCF4), lymphoid enhancer‐binding factor 1 (LEF1), SMAD4, SMAD5, early growth response 1 (EGR1), specificity protein 1 (SP1), v‐jun avian sarcoma virus 17 oncogene homolog (JUN), and v‐myc avian myelocytomatosis viral oncogene homolog (MYC). The temperature profile consisted of initial denaturation for 20 sec at 95°C, followed by 40 cycles of denaturation at 95°C for 3 sec, annealing and elongation at 62°C for 30 sec, and fluorescence monitoring at 60°C. The specificity of the amplification reaction was determined by performing a standard curve analysis and a melting curve analysis. Relative signal quantification was achieved by normalizing the signal of each gene to that of the GAPDH gene.

**Table 1:** Oligonucleotide primers and probes used for gene expression analysis by real‐time PCR

Gene	Primer Sequences, 5′‐3′		
	Forward	Reverse	Probe (5′‐FAM, 3′‐BHQ)
VEGF‐R1	GCATGATGGGAATAGGGAGACA	CCAAGGCCCACTTGATCTTTAG	AGGAAAGGGCGCCTACTCTTCAGG
VEGF‐R2	AATGCGGGAGGTTCAATGTG	GGGAAGAACAAAAGGGTAAAATCC	AGCTGTGTGTGGTGTCAAAGTTTCAGG
VE‐cadherin	GAGCCGAGCCATGTGTCTTT	GGTGTGCCTGAGGGTCAGTT	None
Tie2	CTGTATACCCTCTGTTTCCCTTTCA	TTGGCAGAGGGCATGTTTTCTC	None
eNOS	CAGCAACGCTACCACGAAGA	TGCGTATGCGGCTTGTCA	ATTTTCGGGCTCACGCTGCG
MMP9	CCCGGAGTGAGTTGAACCA	AGGGCACTGCAGGATGTCA	TGGACCAAGTGGGCTACGTGACCT
ID1	AGAACCGCAAGGTGAGCAA	CCAACTGAAGGTCCCTGATGTAG	TGGAGATTCTCCAGCACGTCATCG
ID2	GGGAGCGAAAACGTTAAAATCA	ATTCACGCTCCACCTTTGAAA	TTGCCCAATCTAAGCAGACTTTGCCTT
FOXM1	GTACCTGGATCTTGGGTTCTTCA	CAAAAAGGACTCTGGCAAGCA	TGCAGGGACCCAGACAAGTGGA
FOXH1	AGGGCTGAGCCTGGAATAGC	ACGGCTGGTGACTGATGGAT	TTCCTAGTCCCCTCTTCTCAGCCCA
HEY1	TGGTGGCCCTGAATCCAT	GGCTCTCTCCTAACTAAATCAGAACTG	TGACCAGCTGCTGGTATCTGCCA
SMAD1	GTTCTGCAGCTGGTTAATTCATGT	CAAGCACTCCATATAACTGGTATTG	CTGTGAGAGCAAATGAATAATTCCTGC
SMAD2	GAGGAAATACATGGCCTTTGATG	CTGGCTTCTCGAGCAGAACAG	TCTGGCGTCTACTGCATTTCCCAG
SMAD3	GTGGCTTTTTGGCTCAGCAT	ACACGCGGCCACTTGTTT	CAGAAACACCAAACCAGGCTGGC
FOSL1	CCACACTCTCCATCACCCTCTT	TGGGTAAAGTGGCACCTTCTG	TGTGATCCACCCAACCCTATCTCCTG
NFkB1	TTGTCCCTCTGCTACGTTCCTATT	TGCTGTGGTCAGAAGGAATGC	TCATTAAAGGTATCACGGTCGCCACC
NRF2	CTCAGCACCTTATATCTCGAAGTTTTC	TGCAGGGAGTATTCACTAGGAGAA	CATGCTACGTGATGAAGATGGAAAACCT
HIF1A	TTGTGGAAGTTTATGCTAATATTGTGTAAC	TCTTGTTTACAGTCTGCTCAAAATATCTT	TAAACCTAAATGTTCTGCCTACCCTGTTGG
EPAS1	GTGACATGTAGGTAGGAAGCACTGA	CCAGAGGTGTCGTCCCTCTTAC	AATAGTGTTCCCAGAGCACTTTGCAACTC
TAL1	CAACTCTTTCGGCCTTTTGG	GTCTTCAGCAGAGGGTCACGTA	TGGGTCTGGCCGTACTTGTGATTTC
RUNX1	CCCCCACCTAGGGTCTATTTG	ACGCACGAATTTTCAGGATGT	TGGCAGTTATTGGGTTTGGTCACAA
c‐MYB	GCCAGTCACTGCCTTAAGAACA	GCAGTAAGTACACCGTCATATCTCAAA	TTGATGCAAGATGGCCAGCACTG
GATA1	GGAAGGATGGTATTCAGACTCGAA	GACTGGAGCCCCGTTTCTTT	CGCAAGGCATCTGGAAAAGGGA
GATA2	CGTGTCCCGAGCTTAGATTCTG	GCCCATATTGCACTTGGTCACT	CCCAGGAGGAGGATTGTGCTGATG
ERG	CACTGCTTCTCCTAAGCCTTCTG	TCTGCTGCACCTGCTGTCA	ACAGATGTGGCACCTGCAACCC
HHEX	CCACTTAATGGAAAGGCAAAGG	AAATACTCCAAGGCTGCCTGAA	TACCCCAAATCCAGAGGTGCCTACA
GAPDH	GGTGGTCTCCTCTGACTTCAACA	GTGGTCGTTGAGGGCAATG	ACACCCACTCCTCCACCTTTGACG

eNOS, endothelial nitric oxide synthase; VEGF, vascular endothelial growth factor.

### Flow cytometry

Endothelial progenitor cells were washed with cold PBS and were resuspended in PBS with FcR blocking reagent (MACS), 0.2% FBS, and 2 mmol/L EDTA at 4°C for 30 min. They were stained with monoclonal antibodies specific for the following surface antigens: CD34 (Becton Dickinson, San Jose, CA), CD51/61 (integrin αv/β3; Becton Dickinson), CD54 (ICAM1; Becton Dickinson), CD106 (VCAM1; Becton Dickinson), CD117 (c‐Kit; MACS), CD133 (MACS), CD144 (VE‐cadherin; Beckman Coulter, Brea, CA), CD202b (Tie2; R&D), CD309 (VEGF‐R2; R&D), and VEGF‐R1 (R&D). After incubation at 4°C for 30 min they were washed twice and were analyzed using two‐color flow cytometry.

### Statistical analysis

All results are expressed as means ± SD. Statistical significance was evaluated by ANOVA and a Bonferonni adjustment applied to the results of a *t* test performed with SPSS software. *P*‐values of <0.05 were regarded as statistically significant.

## Results

### Culture of circulating EPCs in dextran

**Figure 1:**
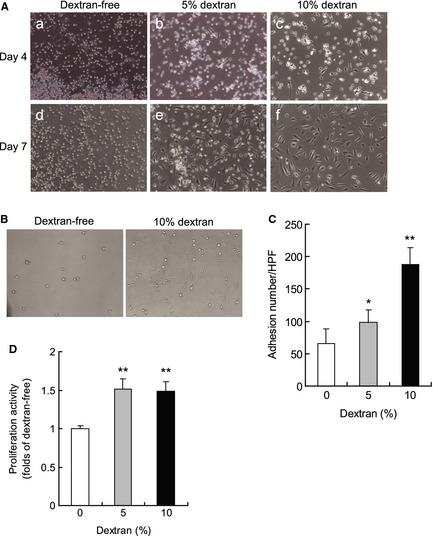
Effect of dextran on the culture, adhesion, and proliferation. 3 × 10^4^/cm^2^ floating endothelial progenitor cells (EPCs) were cultured in medium with 5% dextran (A‐b and ‐e) and 10% dextran (A‐c and ‐f) or without dextran (A‐a and ‐d) on human fibronectin‐coated dishes. After 4 days (A‐a, ‐b, and ‐c) and 7 days (A‐d, ‐e, and ‐f) EPCs were observed by a phase contrast microscope (×10) (A). Dextran induced differentiation of circulating EPCs toward adhesive EPCs. Floating EPCs exposed to various densities of dextran for 24 h were cultured for 6 h and the adhesive cells were observed by a phase contrast microscope (×10) (B). EPCs exposed to dextran significantly increased adhesion. The number of adhesive cells per high‐power field (HPF) was counted (C). *N* = 3. Floating EPCs exposed to various density of dextran for 24 h were cultured for 24 h and the proliferation activity was measured (D). Dextran increased proliferation. *N* = 5. Data are means ± SD. **P <* *0.01, *P <* *0.05 versus dextran‐free control.

Floating EPCs were cultured to investigate whether dextran affects the morphological phenotype of EPCs. EPCs without dextran rarely attached to fibronectin‐coated dishes (Fig. 1A). On the other hand, EPCs with 5% dextran began to attach and became elongated at 4 days after seeding. Then, the adhesive EPCs increased at 7 days. Furthermore, adhesive EPCs with 10% dextran increased more exponentially than those with 5% dextran.

### Dextran increases bioactivities of adhesion and proliferation

An adhesion assay was performed to investigate whether dextran affects the adhesion of floating EPCs. The exposure of dextran to floating EPCs for 24 h increased the adhesion number dextran‐dose‐dependently (Fig. 1B and C).

A proliferation assay was performed to investigate whether dextran affects the proliferation of EPCs. The exposure of 5% and 10% dextran to floating EPCs for 24 h significantly increased the proliferation activity than those not treated with dextran (Fig. 1D).

### Dextran increases migration, tube formation, and differentiated EPC colony formation

**Figure 2:**
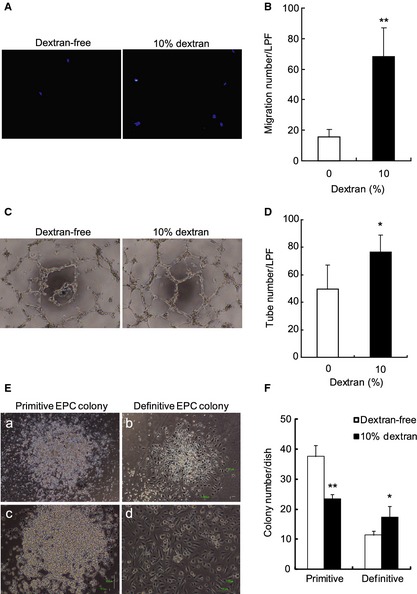
Effect of dextran on the migration, tube formation, and endothelial progenitor cell (EPC) colony formation. Floating EPCs were cultured with or without 10% dextran for 24 h and they were used for measuring following bioactivities. Nuclei of migrated EPCs were stained with DAPI (×10) (A). The number of migrated cells was counted (B). Dextran increased migration. *N* = 3. EPCs under exposure of dextran for 24 h were cultured in matrigel with HUVECs and were observed by a phase contrast microscope (×4) (C). Dextran apparently increased tube formation. The number of tubes per low power field (LPF) was measured (D). *N* = 5. EPCs were cultured in methylcellulose‐containing medium for 15 days, and EPC colonies were observed (E‐a and ‐b, x4; E‐c and ‐d, ×10). Representative pictures of a primitive EPC colony (E‐a and ‐c) and a definitive EPC colony (E‐b and ‐d). Dextran decreased the number of primitive EPC colonies and increased that of definitive EPC colonies (F). *N* = 3. Data are means ± SD. **P <* *0.01, *P <* *0.05 versus dextran‐free control.

A migration assay was performed to study whether dextran affects the migration of EPCs. By using a modified Boyden chamber nuclei of migrated cells were observed (Fig. 2A). The exposure of 10% dextran to EPCs for 24 h increased the migrated number (Fig. 2B).

To investigate whether dextran affects the ability of EPCs to form capillary‐like tubes, a tube formation assay was examined microscopically (Fig. 2C). A quantitative analysis showed that EPCs exposed to 10% dextran for 24 h increased the sum of the tube structure (Fig. 2D).

We have reported that primitive or definitive types of EPC colonies allow us to predict the potential of EPC differentiation (Masuda et al. 2011). The primitive, small cell sized EPC colony has a higher proliferation potential and is composed of immature EPCs meanwhile the definitive, large cell sized EPC colony has more vasculogenic properties and is composed of differentiating EPCs (Fig. 2E). An EPC colony‐forming assay was performed to determine whether dextran affects the endothelial differentiation. The exposure of 10% dextran to EPCs for 24 h decreased the number of primitive EPC colonies, but on the other hand increased that of definitive EPC colonies (Fig. 2F). It indicates that dextran induces endothelial differentiation of circulating EPCs.

### Dextran increases the protein and gene expressions of endothelial markers

**Figure 3:**
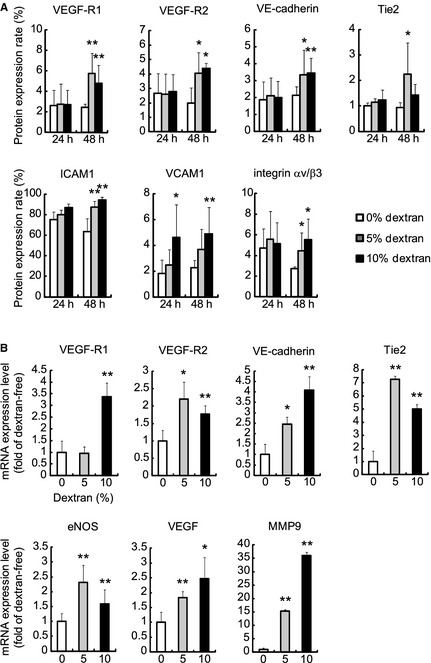
Effect of dextran on the protein and mRNA expression levels of endothelial markers. The expression rates of surface protein in floating endothelial progenitor cells (EPCs) under exposure of 5% and 10% dextran for 24 h (24 h) or 48 h (48 h) were analyzed (A). In 24 h‐EPCs, 10% dextran increased the protein expression of VCAM1. In 48 h‐EPCs, 5% and/or 10% dextran increased vascular endothelial growth factor (VEGF)‐R1, VEGF‐R2, VE‐cadherin, Tie2, ICAM1, VCAM1, and integrin αv/β3. The mRNA expression levels of EPCs under exposure of 5% and 10% dextran for 48 h were analyzed (B). 5% and/or 10% dextran increased gene expression levels of VEGF‐R1, VEGF‐R2, VE‐cadherin, Tie2, endothelial nitric oxide synthase, MMP9, and VEGF. Values are means ± SD of five samples. **P <* *0.01, *P <* *0.05 versus dextran‐free control.

Floating EPCs were exposed to 5% and 10% dextran for 24 and 48 h, and changes in the surface protein expression rate of the endothelial markers VEGF‐R1, VEGF‐R2, VE‐cadherin, and Tie2, and the activated endothelial markers ICAM1, VCAM1, and integrin αv/β3 were analyzed by flow cytometry. The protein expression level of VCAM1 increased in 24 h‐10% dextran EPCs, but others did not change in 24 h‐EPCs (Fig. 3A). Every protein level of 48 h‐EPCs significantly increased in response to 5% and/or 10% dextran.

Floating EPCs were exposed to 5% and 10% dextran for 48 h, and changes in the mRNA levels of the endothelial markers VEGF‐R1, VEGF‐R2, VE‐cadherin, and Tie2, and angiogenic factors eNOS, MMP9, and VEGF were analyzed by real‐time PCR. The expression levels of these genes markedly increased in response to dextran (Fig. 3B).

These findings suggest that dextran increases the surface expression levels of endothelial marker proteins by affecting those genes and induces floating‐circulating EPCs into endothelial differentiation.

### Dextran increases gene expression levels of endothelial cell‐related transcription factors

**Figure 4:**
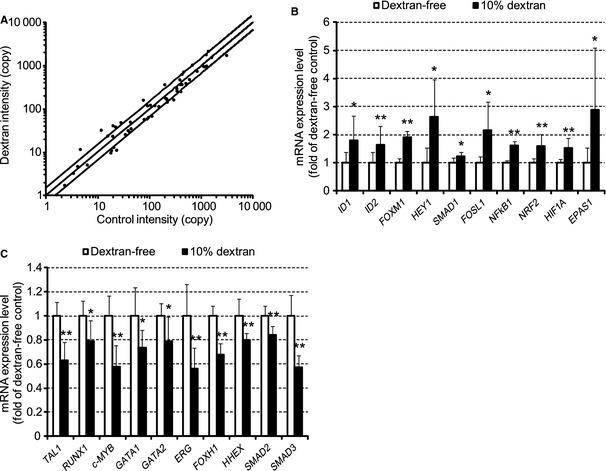
Effect of dextran on the transcription factors. The mRNA expression levels of transcription factors in floating EPCs under exposure of 10% dextran for 48 h were analyzed. Expression levels of 69 genes per 10,000 GAPDH copies are shown in A. The horizontal (*x*) axis indicates copy number in dextran‐free EPCs (control intensity). The vertical (*y*) axis indicates copy number in dextran EPC (dextran intensity). The lines display *y* = 1.5x, *y* = *x*, and *y* = 2/3 *x*, respectively. Relative expression levels of 10 selected genes are shown in B and C. Dextran increased gene expression levels of ID1/2, FOXM1, HEY1, SMAD1, FOSL1, NFkB1, NRF2, HIF1A, and EPAS1. While dextran decreased those of TAL1, RUNX1, c‐MYB, GATA1/2, ERG, FOXH1, HHEX, and SMAD2/3. *N* = 5. Data are means ± SD. **P <* *0.01, *P <* *0.05 versus dextran‐free control.

Floating EPCs were exposed to 10% dextran for 48 h, and 69 representative mRNA expression levels of the transcription factors expressing in embryonic endothelial cells were analyzed by real‐time PCR (Fig. 4A). Thirteen genes in dextran EPCs increased more than 1.5 fold, whereas nine genes in dextran EPCs decreased less than 0.67 fold. Dextran significantly increased the mRNA expression levels of endothelium‐related transcription factors ID1/2, FOXM1, HEY1, SMAD1, FOSL1, NFkB1, NRF2, HIF1A, and EPAS1 (Fig. 4B). On the other hand, dextran significantly decreased those of hematopoietic‐ and anti‐angiogenic‐related transcription factors TAL1, RUNX1, c‐MYB, GATA1/2, ERG, FOXH1, HHEX, and SMAD2/3 (Fig. 4C). These findings indicate that dextran increases both protein and mRNA expression levels of endothelial markers by affecting transcription factors.

### Multiple signal transduction pathways regulate proliferation, adhesion, tube formation, and differentiation

To investigate which signal transduction pathways take part in proliferation, adhesion, tube formation, and differentiation in response to dextran, inhibitors of signal transduction pathways were added in those assays. LY294002, PD98059, JNK inhibitor II, and SB203580 were used as the specific inhibitors of phosphoinositide 3‐kinase (PI3K), extracellular signal‐regulated kinase 1/2 (ERK1/2), c‐Jun N‐terminal kinase (JNK), and p38 mitogen‐activated protein kinase (p38), respectively.

**Figure 5:**
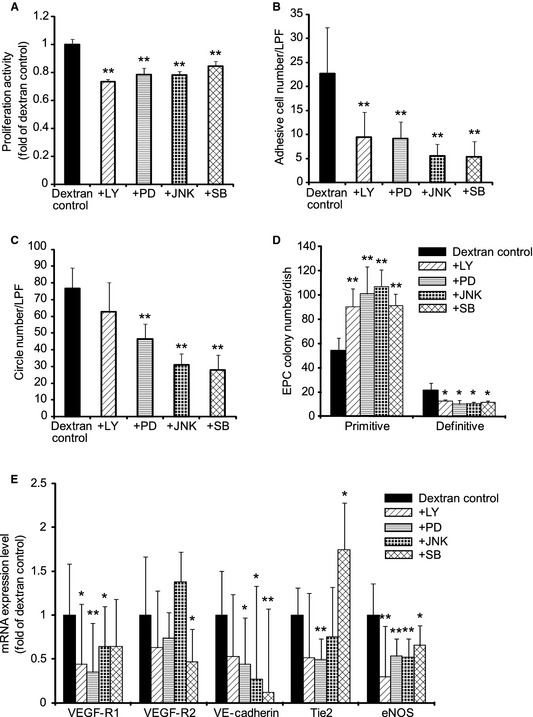
Inhibitor analysis of the adhesion, proliferation, tube formation, endothelial progenitor cell (EPC) colony formation, and differentiation. The abilities of proliferation (A), adhesion (B), tube formation (C), and EPC colony formation were analyzed (D) after floating EPCs were exposed to 10% dextran for 24 h with various inhibitors of signal transduction pathways,. All inhibitors decreased proliferation and adhesion. PD98059, JNK inhibitor II, and SB203580 decreased tube formation. Every inhibitor decreased definitive EPC colony formation, meanwhile, increased primitive EPC colony formation. EPCs were exposed to 10% dextran for 48 h with various inhibitors and the mRNA expression levels were analyzed (E). Inhibitors decreased almost all mRNA expression levels of vascular endothelial growth factor (VEGF)‐R1, VEGF‐R2, VE‐cadherin, Tie2, and endothelial nitric oxide synthase. However, SB203580 increased the mRNA expression level of Tie2. LY, LY294002; PD, PD98059; JNK, JNK inhibitor II; and SB, SB203580. Values are means ± SD of 3–5 samples. **P <* *0.01, *P <* *0.05 versus dextran control.

A proliferation assay and an adhesion assay with inhibitors showed that every inhibitor decreased the proliferation activity and the adhesive cell number (Fig. 5A and B). These results indicate that PI3K/Akt, ERK1/2, JNK, p38 pathways increase bioactivities of proliferation and adhesion in response to dextran.

A tube formation assay showed that the ERK1/2, JNK, p38 inhibitors suppressed tube formation, whereas the PI3K inhibitor did not change it significantly (Fig. 5C). This suggests that ERK1/2, JNK, and p38 pathways increase the dextran‐responsive tube formation.

A colony assay indicated that every inhibitor increased the number of primitive EPC colonies; on the other hand, it decreased the number of definitive EPC colonies (Fig. 5D). This means that PI3K/Akt, ERK1/2, JNK, and p38 are involved in the dextran‐inducing differentiation.

To confirm how signal transduction pathways regulate differentiation of EPCs, EPCs with inhibitors were exposed to 10% dextran for 48 h and endothelial marker genes were analyzed. A real‐time PCR analysis showed that the PI3K inhibitor decreased mRNA expression levels of VEGF‐R1 and eNOS (Fig. 5E). The ERK1/2 inhibitor decreased those of VEGF‐R1, VE‐cadherin, Tie2, and eNOS. The JNK inhibitor decreased those of VEGF‐R1, VE‐cadherin, and eNOS. The p38 inhibitor decreased those of VEGF‐R2, VE‐cadherin, and eNOS, on the other hand, increased that of Tie2. These results indicate that PI3K/Akt, ERK1/2, JNK, and p38 pathways complicatedly regulate the EPC differentiation in response to dextran.

## Discussion

We have developed an epoch‐making EPC differentiation assay. The results of this study showed that dextran enlarged the bioactivities of adhesion, migration, proliferation, and tube formation, as the mRNA expression levels of angiogenic factors, eNOS, MMP9, and VEGF genes increased in floating EPCs cultured in a suspended manner. In addition, dextran increased both protein and gene expression levels of the endothelial markers VEGF‐R1, VEGF‐R2, VE‐cadherin, and Tie2, and activated endothelial markers ICAM1, VCAM1, and integrin αv/β3. Dextran increased differentiating definitive type of EPC colony‐forming cells instead of primitive EPC colony‐forming cells. These findings indicate that dextran induces circulating EPCs toward mature adhesive EPCs.

Dextran has various influences on cell bioactivities through changing osmolality and viscosity, and binding with macrophage mannose receptor (MMR). In addition, dextran may be capable of presenting scaffold and differentiation‐related molecules to cells. There are integrins, the cytoskeleton, receptor tyrosine kinases, and transient receptor potential (TRP) channels which sense osmolality and transmit the information into inner cells (Pedersen et al. 2011). Cell swelling increases integrin β1 followed by Rho activity. The actin cytoskeleton reorganizes rapidly using Rho family G proteins, nonmuscle myosin II, cortactin, and the WASP/Arp2/3 system in response to osmotic stress. Hypotonic swelling activates epidermal growth factor receptor with integrin. TRPV4 displays hypotonicity‐induced calcium influx. However, the change in the medium osmolality in this study by 10% dextran is less than 5% and the values of osmolality are within physiological state. Viscosity is generated by macrorheological parameters, hematocrit and serum proteins like fibrinogen and globulins, and microrheological parameters, the degree of red blood cells aggregation and deformability under blood‐ and tissue‐flow condition. Impairment of responses to viscosity leads to the development of various vascular diseases such as hypertension (Koenig et al. 1989), diabetes (Skovborg et al. 1966; Cho et al. 2008), strokes, and ischemic heart diseases (Lowe et al. 1997). The blood viscosity is about 3.5 cP (Ercan et al. 2002; Lo Presti et al. 2002; Marcinkowska‐Gapińska and Kowal 2006) and the viscosity in bone marrow is 37.5 cP (Gurkan and Akkus 2008). In this study, the viscosity of control medium, medium with 5% dextran, and medium with 10% dextran is about 1, 3.5, and 8 cP, respectively. The viscosity change by 5% or 10% dextran is not likely significant for the physiological environment in which EPCs exist. MMR is expressed in the endothelial cells in embryo and adult, macrophages, and dendritic cells (Sallusto et al. 1995; Takahashi et al. 1998; Gröger et al. 2000). MMR is a 175 kDa transmembrane glycoprotein characterized by a cysteine‐rich NH_2_‐terminal domain, eight C‐type lectin carbohydrate recognition domains with broad specificity for sugars, and a cytoplasmic tail related to endocytosis and phagocytosis (Ezekowitz et al. 1990; Stahl 1992; Taylor and Drickamer 1993). MMR‐knockout mice show that high amounts of mannose and N‐acetylglucosamine reside in serum and that elevated levels of lysosomal hydrolases exist in serum, suggesting that MMR regulates serum glycoprotein homeostasis (Lee et al. 2002). MMR is expressed in M2 macrophages which secrete cytokines including interleukin 10, chemokine, cc motif, ligand 17 (CCL17), CCL22, transforming growth factor beta (TGFβ) and promote tissue repair and angiogenesis (Fairweather and Cihakova 2009). This study shows that dextran increases protein expression levels of integrin αv/β3 in floating‐circulating EPCs. This suggests that EPCs may recognize dextran as scaffold. Furthermore, dextran may serve more differentiation‐related molecules into EPCs. Considering the above‐mentioned, elucidation of the stimulation mechanism by dextran would provide deeper insight into the mechanism of bioactivities and differentiation of EPCs.

There are some EPC studies reporting that transcription factors such as SP1, ID1, HIF1A, FOXO3A, KLF4 affected differentiation of EPCs. We have previously demonstrated that shear stress increased the expression level of arterial marker ephrinB2 by activating SP1 in adhesive EPCs (Obi et al. 2009). Conditional ID1 suppression in EPCs impaired the mobilization of EPCs and angiogenesis (Mellick et al. 2010). Knockdown of HIF1A decreased the expression of VEGF, CD31, VEGF‐R2, and eNOS and the production of NO in adhesive EPCs (Jiang et al. 2006). Expression of FOXO3A was down regulated in differentiated adhesive EPCs, while overexpression of FOXO3A reduced the number of differentiated adhesive EPCs (Mogi et al. 2008). Overexpression of KLF4 in adhesive EPCs increased CD34 expression and decreased tube formation (Li et al. 2012a).

This study showed that dextran increased mRNA expression levels of ID1/2, FOXM1, HEY1, SMAD1, FOSL1, NFkB1, NRF2, HIF1A, and EPAS1 in circulating EPCs. However, dextran decreased those of hematopoietic‐ and anti‐angiogenic‐related transcription factors, such as TAL1, RUNX1, c‐MYB, GATA1/2, ERG, FOXH1, HHEX, and SMAD2/3.

ID1 increases proliferation, migration, and tube formation through transcriptional activation of VEGF by stabilizing HIF1A protein (Lee et al. 2006). ID1 also increases adhesion and tube formation through integrin β and Rho kinase signaling (Qiu et al. 2011). ID1 and ID3 double knockout mice show vascular malformations indicating that ID regulates vascular differentiation (Lyden et al. 1999). FOXM1 increases proliferation, migration, and angiogenesis by inducing VEGF and MMP9 (Ahmad et al. 2010). FOXM1 knockout mice display defects in the formation of peripheral pulmonary capillaries (Kim et al. 2005). HEYs function as downstream targets of arterial endothelium marker Notch signaling pathway and HEY1 is induced by bone morphogenetic protein (BMP) and Notch signaling pathway (Itoh et al. 2004). SMADs function as downstream targets of TGFβ and BMP signaling pathways. SMAD1 and SMAD5 lead to ID1 expression and induce proliferation, migration, and tube formation. While, SMAD2 and SMAD3 lead to plasminogen activator inhibitor 1 expression and inhibit proliferation, migration, and tube formation (Scharpfenecker et al. 2009). FOSL1 knockout mice lack a properly vascularized labyrinth layer of placentas (Schreiber et al. 2000). NFkB is a master regulator of inflammation‐related gene expression such as ICAM1 and VCAM1. It is reported that ID1/PI3K/Akt/NFkB/survivin signaling pathway increases proliferation of EPCs (Li et al. 2012b). NRF2 regulates gene expressions of antioxidant and anti‐inflammation (Mann et al. 2007). HIF1A and EPAS1 are the key factors of angiogenesis in a low oxygen environment although there are many reports in which HIF1A is regulated through oxygen‐independent factors including interleukin 1 beta, TGFβ1, insulin‐like growth factor 2 (Zelzer et al. 1998; Görlach et al. 2001; Jung et al. 2003). TAL1, RUNX1, c‐MYB, GATA1/2, and ERG are representative markers of the HSC lineage (Doré and Crispino 2011). FOXH1 and HHEX inhibit the transcription of VEGF‐R2 and suppress angiogenesis (Minami et al. 2004; Choi et al. 2007).

Taken together, these transcription factors are important for EPC differentiation. Further studies of interaction among these transcription factors will elucidate the differentiation process and the origin of EPCs as well as developmental endothelial cells.

**Figure 6:**
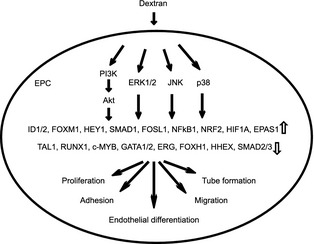
A schematic diagram which shows that dextran induces differentiation of circulating endothelial progenitor cells.

Previous studies have reported that the PI3K/Akt signaling pathway regulates the differentiation of circulating EPCs; mechanical shear stress induces endothelial differentiation of circulating EPCs via the PI3K/Akt/mTOR pathway (Obi et al. 2012), and ginsenoside Rg3 decreases differentiation of circulating EPCs via the Akt/eNOS pathway (Kim et al. 2012). This study showed that dextran induced differentiation of circulating EPCs toward adhesive EPCs through multiple signal transductions including PI3K/Akt, ERK1/2, JNK, and p38 (Fig. 6). It is reported that dextran binds with MMR in dendritic cells and is taken up by endocytosis via the mTOR, JNK, and p38 signaling pathways (Arrighi et al. 2001; Hackstein et al. 2002; Nakahara et al. 2004). Clarification of the differences in these pathways would lead to a better understanding of the molecular mechanism by which dextran regulates differentiation of circulating EPCs.

Many studies have demonstrated that circulating EPCs are influenced by physiological and pathological factors such as age, estrogen, exercise, smoking, hypertension, hyperlipidemia, diabetes mellitus, myocardial ischemia, heart failure, and renal failure (Leone et al. 2009). Circulating EPCs are also influenced by drugs including angiotensin‐converting enzyme inhibitor, hydroxymethylglutaryl‐CoA reductase inhibitor, peroxisome proliferator‐activated receptor γ, insulin, erythropoietin, granulocyte colony‐stimulating factor (G‐CSF). These factors affect mobilization, homing, adhesion, migration, proliferation, and vasculogenesis. Clinically G‐CSF is used by EPC transplantation therapy for myocardial ischemia and hind limb ischemia (Li et al. 2007; Losordo et al. 2007; Kawamoto et al. 2009; Lara‐Hernandez et al. 2010). This study showed that dextran increased bioactivities of proliferation, adhesion, migration, and tube formation. It suggests that dextran may be effective for the EPC‐mediated therapy.

In conclusion, we have made an EPC differentiation assay by using dextran. Dextran increases differentiation, adhesion, migration, proliferation, and vasculogenesis of circulating EPCs. The differentiation mechanism in response to dextran is regulated by multiple signal transductions including PI3K/Akt, ERK1/2, JNK, and p38. The new differentiation assay using dextran will clarify the molecular and physiological mechanisms at successive stages of EPC differentiation from circulation to tissue.

## Conflict of Interest

None declared.
